# Intermittent fasting: is there a role in the treatment of diabetes? A review of the literature and guide for primary care physicians

**DOI:** 10.1186/s40842-020-00116-1

**Published:** 2021-02-03

**Authors:** Michael Albosta, Jesse Bakke

**Affiliations:** 1 Central Michigan University College of Medicine, 1200 S. Franklin St., Mount Pleasant, MI 48858 USA; 2 Saginaw, MI 48602 USA

**Keywords:** Diabetes, Intermittent fasting, Health, Nutrition

## Abstract

**Background:**

Type 2 Diabetes is a metabolic disorder characterized by hyperglycemia that causes numerous complications with significant long-term morbidity and mortality. The disorder is primarily due to insulin resistance particularly in liver, skeletal muscle, and adipose tissue. In this review, we detail the hormonal mechanisms leading to the development of diabetes and discuss whether intermittent fasting should be considered as an alternative, non-medicinal treatment option for patients with this disorder.

**Methods:**

We searched PubMed, Ovid MEDLINE, and Google Scholar databases for review articles, clinical trials, and case series related to type 2 diabetes, insulin resistance, and intermittent fasting. Articles were carefully reviewed and included based on relevance to our topic. We excluded abstracts and any non-English articles.

**Results:**

The majority of the available research demonstrates that intermittent fasting is effective at reducing body weight, decreasing fasting glucose, decreasing fasting insulin, reducing insulin resistance, decreasing levels of leptin, and increasing levels of adiponectin. Some studies found that patients were able to reverse their need for insulin therapy during therapeutic intermittent fasting protocols with supervision by their physician.

**Conclusion:**

Current evidence suggests that intermittent fasting is an effective non-medicinal treatment option for type 2 diabetes. More research is needed to delineate the effects of intermittent fasting from weight loss. Physicians should consider educating themselves regarding the benefits of intermittent fasting. Diabetic patients should consult their physician prior to beginning an intermittent fasting regimen in order to allow for appropriate oversight and titration of the patients medication regimen during periods of fasting.

**Supplementary Information:**

The online version contains supplementary material available at 10.1186/s40842-020-00116-1.

## Introduction

Type 2 Diabetes Mellitus (DM) is a common metabolic disorder characterized by hyperglycemia caused by various factors including impaired insulin secretion, insulin resistance, decreased glucose utilization, excessive hepatic glucose production, and systemic low-grade inflammation [1]. According to the CDC, diabetes affects 34.2 million people in the United States (10.5% of the total population) [2]. Diabetes is known to be responsible for the development of multiple long term complications, which contribute to the disease’s morbidity and mortality. For instance, diabetes is the leading cause of renal failure, new onset blindness, and nontraumatic lower extremity amputation in the United States [3]. The complications of diabetes can be either vascular or non-vascular in nature. The vascular complications include retinopathy, macular edema, mono- and polyneuropathy, autonomic dysfunction, nephropathy, coronary heart disease, peripheral vascular disease and stroke [3]. Non-vascular complications include issues with the gastrointestinal tract (gastroparesis), changes in skin color, increased risk of infections, cataracts, glaucoma, periodontal disease, and hearing loss [3]. Currently the goal of treatment for type 2 diabetes is centered around preventing or delaying complications and maintaining quality of life for the patient, as described by a consensus report for the management of hyperglycemia by the American Diabetes Association (ADA) and European Association for the Study of Diabetes (EASD) [4]. While it is encouraged that patients with type 2 diabetes engage in lifestyle changes including increased physical activity, weight loss, and medical nutrition therapy, a majority of patients require the use of medications to achieve control of their blood glucose levels [5]. Although it has been well described that type 2 diabetes is a disease of insulin resistance, a large amount of the medical therapies that physicians use are based around the premise of giving the patient more insulin. For instance, drugs like the sulfonylureas, GLP-1 agonists, DPP-4 inhibitors, and various insulin preparations all work by either increasing the endogenous production of insulin or increasing the amount of exogenous insulin received. While this works to reduce hyperglycemia in these patients, the idea of treating a disease of insulin resistance by increasing insulin may be counterproductive, leading to the requirement of increasing amounts of medication over a long period of time. In fact, a study by Henry et al. [6] showed that when treating type 2 diabetics with intensive insulin therapy to achieve tight glycemic control, the patients all developed increased hyperinsulinemia and weight gain over a 6 month period.

**Fig. 1:**
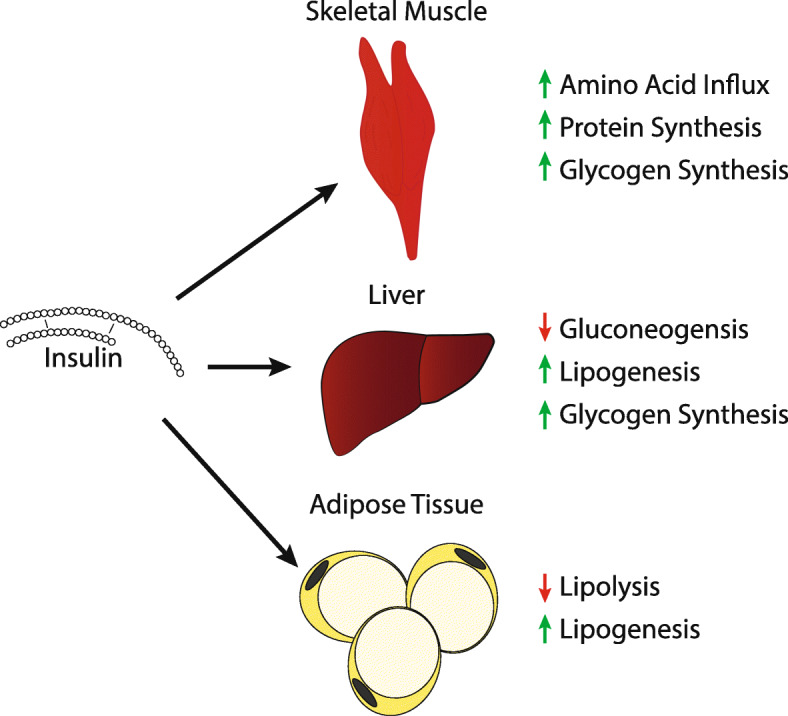
Effects of Insulin on Various Tissues [7, 8]

**Fig. 2:**
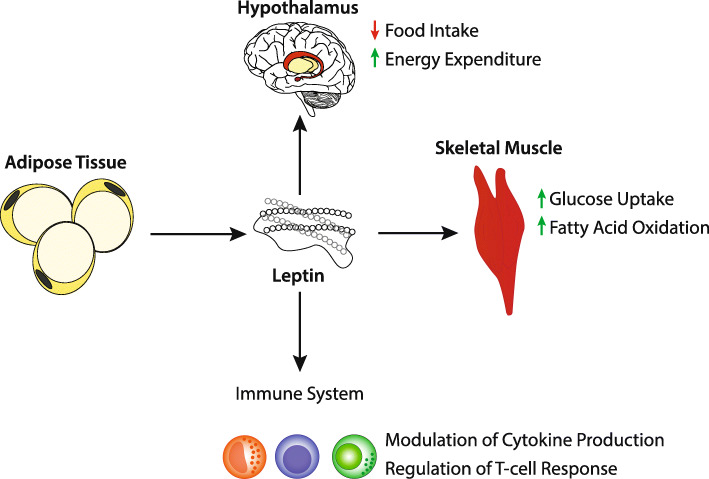
Effects of Leptin [9, 10]

**Fig. 3:**
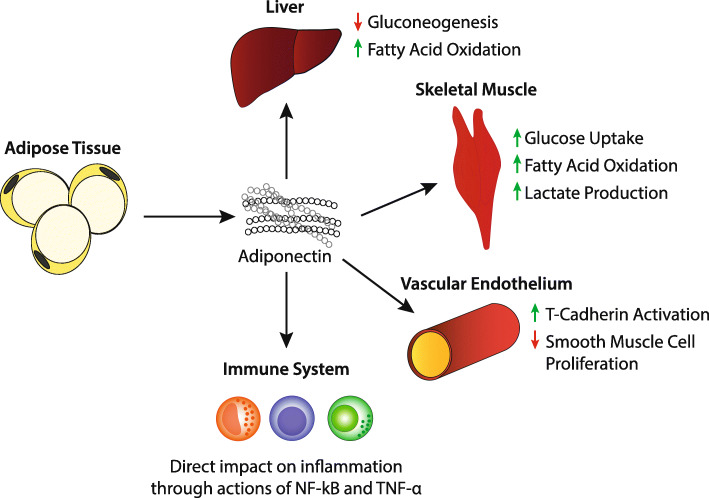
Effects of Adiponectin [10]

Although the ADA and EASD describe the goal of treatment as being aimed at preventing or delaying the complications of this disease, the goal of this review is to take a closer look at the possibility of using intermittent fasting as a non-medicinal option for the treatment of type 2 diabetes through improved insulin sensitivity. When considering the therapeutic role of intermittent fasting in patients with diabetes, there are three hormones that likely play a significant role. These include insulin, as well as the adipokines leptin and adiponectin. Figures 1, 2 and 3 describe the effects of these hormones on various tissues. It is the purpose of this review to provide insight into the influence of these hormones on the development of insulin resistance and type 2 diabetes, as well as the beneficial effects of intermittent fasting on these metabolic markers. Moving forward, we hope this review is a summary of the current literature on the use and efficacy of intermittent fasting in the clinic. We also hope this review serves as a catalyst for physicians to publish case reports and partake in controlled studies regarding intermittent fasting and diabetes.

## Methods

A literature review was performed for articles related to the impact of intermittent fasting on type 2 diabetes mellitus. We used PubMed, Google Scholar, and Ovid MEDLINE to search for published articles, including randomized controlled trials, clinical trials, case reports, and case series between the years of 1990 and 2020. Searches through the references of retrieved articles was also performed. Finally, the websites of professional organizations such as the American Diabetes Association and European Association for the Study of Diabetes were searched for specific guidelines and recommendations. The following keywords were used: “intermittent fasting”, “type 2 diabetes mellitus”, “diabetes”, “fasting”, “obesity”, “hormones”, “insulin”, “leptin”, “adiponectin”, “insulin resistance”. Inclusion criteria consisted of published articles, articles that were available in English, and trials/reports with human subjects. Trials were included if the study design involved one of the three most commonly reported intermittent fasting regimens: alternate day fasting, periodic fasting, or time-restricted feeding. Finally, studies were included if the outcome measures included measurement for fasting glucose, HbA1C, fasting insulin, leptin, or adiponectin both in patients with and without a history of diabetes. Exclusion criteria consisted of duplicates, abstracts, non-English articles, articles that did not include human subjects, those that did not report outcome measures for any of the previously described variables, and works that were unpublished or unrelated to the topic of interest. Our initial search returned 6852 studies. Two reviewers independently reviewed abstracts to determine whether studies met our inclusion criteria. Studies that met criteria were then further reviewed to determine whether they would be included in our review. After careful review, a total of 17 articles were ultimately chosen and are available for review in Tables 1 and 2.

**Table 1:** Clinical Trials of Fasting Regimens in Patients with and without Diabetes

***Intermittent Fasting Protocol***	***Description***	***Effects of Intermittent Fasting*** vs. ***Baseline***	
***Alternate Day Fasting***	***One day of fasting/One day of*** **ad libitum** ***feeding***	***Trepanowski*** **et al** [11]. ***(6 months)***	***Average Baseline Values*** Weight: 96 kg Fasting Glucose: 90 mg/dL Fasting Insulin: 18 uIU/mL HOMA-IR: 4.1 ***Results*** - Decreased body weight by 6.8% - Decreased fasting glucose by 6.3% - Decreased fasting insulin by 7.5% - Decreased HOMA-IR by 2.49%
		***Catenacci*** **et al.** [12] ***(8 weeks)***	***Average Baseline Values*** Weight: 94.7 kg Fasting glucose: 88.4 mg/dL Leptin: 29.8 ng/mL ***Results*** - Decreased body weight by 8.2 kg - Decreased glucose by 6.0 mg/dL - Decreased leptin by 13.9 ng
		***Bhutani*** **et al.** [13] ***(12 weeks)***	***Average Baseline Values*** Body weight: 94 kg Fasting Glucose: 98 mg/dL Fasting insulin: 21.8 uIU/mL ***Results*** - Decreased body weight by 3 kg - Decreased fasting glucose by 3% - Decreased fasting insulin by 7%
		***Bhutani*** **et al.** [14] ***(8 weeks)***	***Average Baseline Values:*** Body weight: 96.4 kg Leptin and Adiponectin (see study) ***Results:*** - Decreased body weight by 5.7 kg - Decreased leptin by 21% - Increased adiponectin by 30%
		***Varady*** **et al.** [15] ***(12 weeks)***	***Average Baseline Values:*** Body weight: 77 kg Adiponectin: 10728 ng/mL Leptin: 25 ng/mL ***Results*** - Decreased body weight by 5.2 kg - Increased adiponectin by 0.7μg/ml - Decreased leptin by 10 ng/ml
		***Gabel*** **et al.** [16] ***(12 months)***	***Average Baseline Values*** Body weight: 95 kg Fasting Glucose: 99 mg/dL Fasting Insulin: 23 uIU/mL ***Results:*** ***-*** Decreased body weight by 8% - Decreased fasting insulin by 52% - Decreased Fasting glucose by 3%
		***Hoddy*** **et al.** [17] ***(10 weeks)***	***Average Baseline Values*** Body Weight: 94 kg/97 kg (timing of fasting day single low calorie meal: Lunch/Dinner) BMI: 35/34 kg/m^2^ Fasting Glucose: 96/100 mg/dL Fasting insulin: 12/11 uIU/mL HOMA-IR: 3.0/3.0 ***Results*** - Body weight decreased by 3.5/4.1 kg - BMI reduced by 1.3/1.4 kg/m^2^ - Fasting Glucose, Insulin, and HOMA-IR did not undergo statistically significant changes
***5/2 method***	***Complete fasting on 2 non-consecutive days/5 days of*** **ad libitum** ***feeding***	***Carter*** **et al .**[18] ***(12 weeks)***	***Average Baseline Values:*** HbA1C: 7.2% Body Weight: 99 kg ***Results:*** - Decreased HbA1C by 0.7% - Decreased body weight by 8 kg
		***Carter*** **et al.** [19] ***(12 months)***	***Average Baseline Values:*** HbA1C: 7.2% Body Weight: 100 kg ***Results*** - Decreased body weight by 6.8 kg - Decreased HbA1C by 0.3%
		***Sundfor*** **et al.** [20] ***(6 months)***	***Average Baseline Values:*** Body Weight: 108.6 kg HbA1C: 5.6% ***Results:*** - Decreased body weight by 9.1 kg - Decreased HbA1C by 0.3%
		***Corley*** **et al.** [21] ***(12 weeks)***	***Average Baseline Values (Consecutive/Non-consecutive 2 day fast)*** Weight: 108.7/109.8 kg BMI: 36.6/36.8 kg/m^2^ HbA1C: 8.4/8.2% ***Results (Consecutive/Non-consecutive 2 day fast)*** - Decreased weight by 3.1/3.6 kg - Decreased BMI by 0.5/0.8 kg/m^2^ - Decreased HbA1C by 0.6/0.7%
***Time Restricted Feeding***	***Daily 12–20 h fast/4–12 h feeding window***	***Moro*** **et al.** [22] ***(8 weeks)***	***Average Baseline Values:*** Fat mass: 10.90 kg Fasting glucose: 96.64 mg/dL Fasting insulin: 2.78 mU/mL Adiponectin: 11.8 μg/mL Leptin: 2.1 ng/mL ***Results:*** - Decreased fat mass by 1.62 kg - Decreased fasting glucose by 10.72 mg/dL - Decreased fasting insulin by 1.01 mU/mL - Increased adiponectin by 2.1 μg/mL - Decreased leptin by 0.3 ng/mL
		***Sutton*** **et al.** [23] ***(5 weeks)***	***Average Baseline Values:*** Insulin: 25.1 mU/L Insulinogenic Index: 113 U/mg Glucose: 100 mg/dL ***Results*** - Decreased fasting insulin by 0.4 mU/L - Increased insulinogenic index (marker of Beta cell responsiveness) by 14 U/mg - No significant change in fasting glucose
		***Hutchison*** **et al.** [24] ***(1 week)***	***Average Baseline Values:*** Weight: 105.7 kg BMI: 33.9 kg/m^2^ Fasting Glucose: 5.8 mmol/L ***Results:*** - Decreased glycemic response to test meal by 36% - No significant weight reduction
		***Cienfuegos*** **et al.** [25] ***(8 weeks) (4 h feeding window)***	***Average Baseline Values:*** Body Weight: 101 kg Fasting Glucose: 88 mg/dL Fasting insulin: 12 uIU/mL HOMA-IR: 2.7 HbA1C: 5.9% ***Results:*** - Body weight decreased by 3.2% - Fasting glucose decreased by 5.0 mg/dL - Fasting insulin decreased by 2.3 uIU/mL - HOMA-IR decreased by 29% - HbA1C decreased by 0.2%
		***Cienfuegos*** **et al.** [25] ***(8 weeks) (6 h feeding window)***	***Average Baseline Values:*** Body Weight: 99 kg Fasting Glucose: 94 mg/dL Fasting insulin: 16 uIU/mL HOMA-IR: 3.7 HbA1C: 5.9% ***Results:*** - Body weight decreased by 3.2% - Fasting glucose decreased by 2.3 mg/dL - Fasting insulin decreased by 1.9 uIU/mL - HOMA-IR decreased by 12% - HbA1C decreased by 0.2%

**Table 2:** Case series/reports investigating intermittent fasting in patients with type 2 diabetes

***Intermittent Fasting Protocol***	***Description***	***Effects of Intermittent Fasting*** vs. ***Baseline***	
***Alternate Day Fasting***	***One day of fasting/One day of*** **ad libitum** ***feeding***	***Furmli*** **et al.** [26] ***(7 months)***	***Average Baseline Values:*** ***HbA1C: 11%, 96.7 mmol/mol*** ***Body weight: 83.8 kg*** ***Results:*** ***- Decreased HbA1C by 4%*** ***- Decreased body weight by 10 kg***
		***Furmli*** **et al.** [26] ***(11 months)***	***Average Baseline Values:*** ***HbA1C: 7.2%, 55.2 mmol/mol*** ***Body weight: 61 kg*** ***Results:*** ***- Decreased HbA1C by 0.8%*** ***- Decreased body weight by 9 kg***
		***Furmli*** **et al.** [26] ***(11 months)***	***Average Baseline Values:*** ***HbA1C: 6.8%, 50.8 mmol/mol*** ***Body weight: 97.1 kg*** ***Results:*** ***- Decreased HbA1C by 1.2%*** ***- Decreased body weight by 10.6 kg***
		***Lichtash*** **et al.** [27] ***(14 months)***	***Average Baseline Values*** ***HbA1C: 9.3%*** ***Body Weight: 55.3 kg*** ***Results:*** ***- Decreased HbA1C by 3.5%*** ***- Decreased body weight by 1.8 kg***

### What is intermittent fasting?

**Fig. 4:**
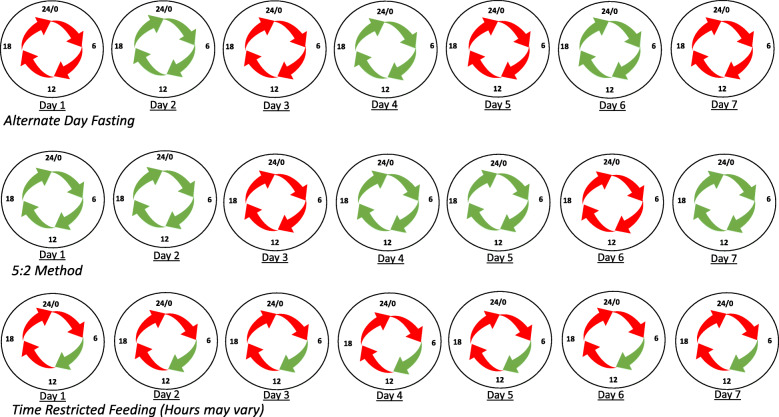
Intermittent Fasting Regimens

Intermittent fasting has recently gained popularity as a means of improving body composition and metabolic health [28, 29]. Intermittent fasting refers to eating patterns based around the principle of consuming very little to no calories for time periods ranging from 12 h to several days with a regular pattern [28]. There are several different regimens of intermittent fasting. One such regimen is alternate day fasting, in which days of fasting are separated by days of ad libitum food consumption [29]. Another method is periodic fasting, in which individuals fast for 1 or 2 days a week (also referred to as 5:2 or 6:1 fasting) [29]. Finally, the most common method is time-restricted feeding, in which food consumption is only allowed during a specified window of time each day, typically with 16–20 h daily fasts [29]. See Fig. 4 for a visual representation of the most common intermittent fasting regimens.

### Intermittent fasting, weight loss, and appetite control

Obesity is known to be a major risk factor for the development of type 2 DM. There are a number of mechanisms believed to contribute to the development of insulin-resistance in obese patients. These include, but are not limited to, systemic chronic inflammation and ectopic lipid deposition [7, 9, 30, 31]. Visceral adipose tissue is known to function as both a paracrine and endocrine organ through the secretion of adipokines [10]. These adipokines are either proinflammatory leading to chronic low-level inflammation, such as leptin, or anti-inflammatory such as adiponectin [10]. Leptin is known to play a role in the regulation of body weight through signaling to the hypothalamus and other brain regions to suppress food intake and increase energy expenditure [9]. The inflammatory effects of leptin are likely due to its role in the production of IL-6, which induces the synthesis of C-reactive protein in the liver as well as upregulation of the pro-inflammatory cytokine TNF-alpha [10]. Interestingly, patients with higher levels of BMI and insulin resistance were found to have increased leptin levels, possibly signifying that patients with obesity and insulin resistance are developing leptin resistance as well [10]. On the contrary, adiponectin is known to have antidiabetic and anti-inflammatory effects. Adiponectin acts on various receptors that results in an increase in skeletal muscle and hepatic fatty acid oxidation, reduced hepatic gluconeogenesis, and increased glucose uptake [10]. It also exerts anti-inflammatory effects through direct action on inflammatory cells, action of NF-kB, and interactions with TNF-alpha [10]. Adiponectin levels decrease with accumulation of visceral fat [10]. López-Jaramillo et al. performed a review with the intention of determining levels of leptin and adiponectin in patients with metabolic syndrome. They found that in patients with the metabolic syndrome, which includes obesity and insulin resistance, an imbalance in levels of leptin and adiponectin appeared to play a role in metabolic alteration that increased the risk of type 2 diabetes [10]. Interestingly, several studies have demonstrated that intermittent fasting, even in the absence of fat loss, has resulted in a reduction of leptin levels and an increase of adiponectin, which results in improvements of insulin resistance [32].

It has long been known that restricting calories can reduce body weight and increase metabolic health [33]. A study by Larson-Meyer et al. [34] showed that 25% calorie reduction either via diet alone or diet in conjunction with exercise led to improvements in insulin sensitivity and reduction in β-cell sensitivity in overweight, glucose-tolerant individuals. However, several obesity trials have demonstrated that humans have significant difficulty sustaining daily calorie restriction for extended periods of time [28]. On the other hand, intermittent fasting has higher compliance and has shown promise in the improvement of metabolic risk factors, body composition, and weight loss in obese individuals [28, 35, 36]. It has been shown that these beneficial effects are due in part to the shift during fasting from the utilization of glucose to fatty acids and ketones as the body’s preferred fuel source [28]. During this transition the body begins to switch from the synthesis and storage of lipids to mobilization of fat in the form of ketone bodies and free fatty acids [28]. This transition of fuel source, or metabolic reprogramming, has been highlighted as a potential mechanism for many of the beneficial effects of intermittent fasting. Lastly, intermittent fasting has been shown to reduce adiposity, particularly visceral fat and truncal fat, largely due to mild energy deficits [12, 17]. It is through this reduction in adiposity that patients may experience improvements in their leptin/adiponectin levels and sensitivity, leading to improved appetite control and lower levels of chronic inflammation thus improving several risk factors for type 2 diabetes.

### Intermittent fasting and insulin sensitivity

Insulin plays a significant role in glucose homeostasis due to its influence in promoting the storage and utilization of glucose. However, the effects of insulin are not limited to glucose homeostasis. Insulin also plays a role in the stimulation of DNA synthesis, RNA synthesis, cell growth and differentiation, amino acid influx, protein synthesis, inhibition of protein degradation, and most importantly, the stimulation of lipogenesis and inhibition of lipolysis [8].

It is the development of insulin resistance, which is defined as the necessity of higher circulating insulin levels in order to produce a glucose lowering response, that is thought to be responsible for the development of type 2 diabetes [7]. In order to promote regulation of glucose homeostasis, insulin works primarily on receptors in skeletal muscle, liver, and white adipose tissue [7]. In short, there are several proposed mechanisms regarding the development of insulin resistance. One of the more prominent theories describes the association of increased adiposity and the subsequent chronic inflammation that leads to the development of insulin resistance in tissues [7].

Intermittent fasting, as described previously, may reduce adiposity and subsequently insulin resistance via reduction of caloric intake as well as due to metabolic reprogramming. In addition, energy/nutrient depletion (such as that achieved through reduced caloric intake) has been shown to promote healthier aging and reduction in chronic disease through increased activation of AMP activated protein kinase (AMPK) [37]. AMPK responds to both to increased AMP/ADP:ATP ratios as well as to endocrine signals of hunger and satiety [37]. The role of AMPK at a biochemical level is outside of the scope of this review, however activation of AMPK through a low energy state has been shown to initiate physiologic responses that promote healthy aging [37]. Increased levels of insulin, whether through increased energy intake or insulin resistance, leads to the activation of downstream mediators that ultimately inhibit AMPK. The role of AMPK in improved insulin sensitivity is most evident via the positive effects of the commonly prescribed biguanide, metformin. Metformin is known to promote the activation of AMPK, and has been shown to be very effective in the treatment of type 2 diabetes as well as in the mitigation of a number of chronic disease states [37]. In theory, decreased energy intake, such as that is achieved through intermittent fasting, will lead to prolonged decreased levels of insulin production and increased levels of AMPK, which likely plays a role in the improvements in insulin sensitivity and glucose homeostasis.

### Intermittent fasting as a treatment for type 2 diabetes?

Several studies have shown promise for the use of intermittent fasting protocols as a potential treatment for diabetes. Tables 1 and 2 illustrate the findings of several recent studies regarding intermittent fasting and its effect on measures including body weight, fasting glucose, fasting insulin, adiponectin, and leptin. The inclusion/exclusion criteria can be found in the supplementary file S1. In a systematic review and meta-analysis by Cho et al. [32] that included studies evaluating patients both with and without pre-diabetes (diabetic patients were excluded), it was found that of 8 studies comparing the effects of an intermittent fasting diet to a control group, BMI decreased by 0.75 kg/m^2^ over periods ranging from 4 to 24 weeks. Furthermore, of 8 studies comparing intermittent fasting to a control group in the evaluation of glycemic control, it was found that the intermittent fasting group had significant reductions in fasting glucose levels (− 4.16 mg/dL; *p* = 0.003). Lastly, when comparing leptin and adiponectin levels between the intermittent fasting subjects and the control subjects in all studies, the reviewers found increased adiponectin levels (1008.87 ng/mL; *p* = 0.023) and decreased leptin (− 0.51 ng/mL; *p* < 0.001) [32]. A case series by Furmli et al. [26] followed three patients with type 2 diabetes over several months after beginning an intermittent fasting regimen consisting of three 24 h fasts per week. Over the course of the study, all patients had significant reductions in HbA1C, weight loss, and all of the patients were able to stop their insulin therapy within 1 month [26]. Interestingly, the three patients in this case series all reported tolerating fasting very well, and no patient stopped the intervention at any point out of choice [26]. This suggests that intermittent fasting may not only be successful as a non-medicinal treatment option for patients with type 2 diabetes, but supports the notion that this intervention is tolerable as well. Carter et al. [19] performed a clinical trial in which 137 adults with type 2 diabetes were divided into two groups, one intermittent energy restriction group (500–600 kcal/day for 2 days per week and normal diet every other day) and a continuous energy restriction group (1200–1500 kcal/day). After 12 months of intervention, the two groups showed similar reductions in HbA1C levels and greater reductions in weight in the intermittent energy restriction group. Finally, a similar clinical trial by Gabel et al. [16] compared an alternate day fasting regimen (25% of energy needs on fasting days, 125% of energy needs on non-fasting days) to continuous energy restriction (75% of energy needs daily) and a control group of obese, non-diabetic patients. Over an intervention period of 12 months, there were similar reductions in body weight, BMI, and fat mass between the alternate day fasting and continuous energy restriction groups, however there were significant reductions in fasting insulin levels (− 44%; *p* < 0.05) and homeostatic model assessment of insulin resistance (HOMA-IR) levels (− 53%; p < 0.05) in the alternate day fasting group [16]. HOMA-IR is a marker used to measure levels of insulin resistance.

### Prescribing intermittent fasting in practice: recommendations

**Fig. 5:**
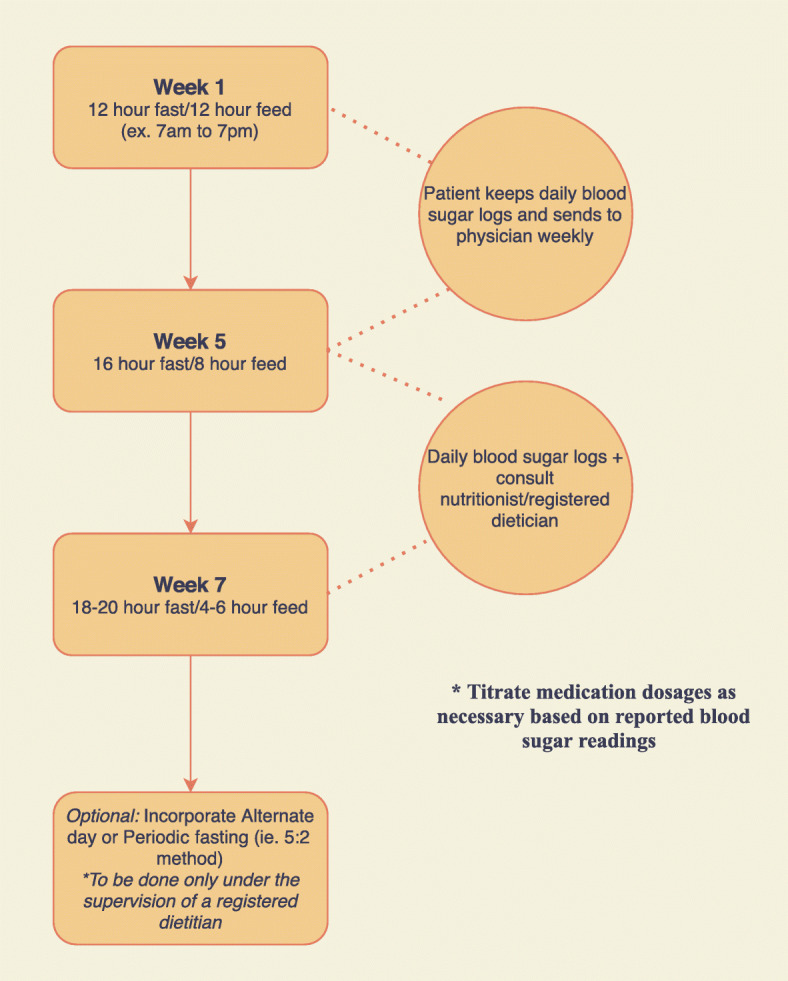
Example of Intermittent Fasting Prescription

While alternate day fasting and periodic fasting have demonstrated efficacy in improving metabolic risk factors, it may be difficult to convince patients to give up or severely restrict calories for an entire 24 h period. In America, we often eat 3 meals per day in addition to frequent snacking. Furthermore, in American culture most social engagements involve food. Asking patients to eliminate these experiences from their day to day lives may become burdensome, and thus hinder patient compliance. Finally, patients switching to an intermittent fasting regimen may initially experience symptoms such as hunger and irritability, although these symptoms often dissipate within the first 30 days [38]. Therefore, it would be more appropriate to gradually introduce intermittent fasting in the form of time restricted feeding. For example, clinicians may first recommend that patients restrict their intake to a daily 12 h period, typically an overnight fast (for example, 7 pm to 7 am). As patients become more comfortable with this pattern of eating, the feeding window can be restricted further (16 h fast followed by an 8 h feed or 20 h fast followed by a 4 h feed). This allows the patient some daily flexibility in choosing when to consume calories, thus increasing the likelihood of compliance. Lastly, patients who have become adapted to time restricted feeding may choose to switch to alternate day or periodic fasting with the supervision and guidance of a registered dietician. See Fig. 5 for a detailed example of an intermittent fasting prescription.

### Prescribing intermittent fasting in practice: considerations

When considering the use of fasting in patients with diabetes, a number of points should be weighed. First, it is important to discuss potential safety risks associated with fasting. Patients taking insulin or sulfonylurea medications should be closely monitored by their healthcare provider in order to prevent hypoglycemic events [39]. Because studies are demonstrating a decreased need for insulin in patients who follow intermittent fasting protocols, blood glucose levels and medication titration should be observed closely by the physician. Physicians should help patients make appropriate adjustments to their medications, especially on days of fasting. Physicians may choose to have patients keep daily blood sugar and weight logs and send them weekly or biweekly via electronic message in order to assist providers in medication titration over time. Of note, while the goal of adapting this pattern of eating is to reduce or eliminate the need for medications, including insulin, there are situations in which insulin may be necessary, such as severe hyperglycemia. Failure to do so may result in significant consequences, such as the development of hyperosmolar hyperglycemic syndrome. Additional concerns, although unlikely, include vitamin and mineral deficiencies and protein malnutrition [39]. Patients should be educated regarding the importance of consuming nutrient-rich meals and adequate protein intake during feeding periods. Furthermore, it may be important to consider vitamin or mineral supplementation depending on the patient’s dietary practices and the desired length of a fasting regimen. Patients should also be counseled on the need for adequate hydration during periods of fasting, as they will be required to replace fluids that might normally be consumed through food in addition to regular daily requirements. As many physicians may not be trained extensively in nutritional sciences, and further, may not have time to follow daily with patients to ensure appropriate nutritional intake, consultation with a registered dietitian is highly recommended. Lastly, it is important to consider populations in whom fasting may not be appropriate. These include pregnant/lactating women, adults of advanced age, individuals with immunodeficiencies, individuals with hypoglycemic events, and patients who suffer from eating disorders [39].

### Limitations and future research

This review is not a systematic review and as such lacks the power to summarize all trails with statistical significance. Having said that, we highlighted the research that has been done in humans and presented evidence that intermittent fasting improves insulin sensitivity, likely through a combination of weight-loss and “metabolic reprogramming”. There is a significant amount of research that has been done on the effects of intermittent fasting in regards to improvements in body composition and metabolic health, however a majority of the data to date has come from animal studies, which were not included in this review. Although there are a number of case reports showing significant improvements in diabetic patients’ glucose control, many of the randomized controlled trials fail to include patients with diabetes. This is an area where further research is needed, as the current trials (and case reports) included in this review that have been done on diabetic patients have shown promise in improving metabolic health with nearly no adverse effects. Most patients doing some form of intermittent fasting experience mild energy deficits and weight-loss, that may not be appropriate for all patients. As such, there needs to be more research into delineating the metabolic improvements of intermittent fasting from weight-loss.

## Conclusion

Type 2 diabetes afflicts 34.2 million people in the United States, and is associated with significant morbidity and mortality [1]. Although diabetes is characterized as a disorder of insulin resistance, a majority of the pharmaceutical treatments for this disease promote increases in insulin levels to achieve better glycemic control. This leads to a number of issues including weight gain, worsened insulin resistance, increased levels of leptin, and decreased levels of adiponectin. Intermittent fasting has become an increasingly popular dietary practice for the improvement of body composition and metabolic health [28, 29]. It also has shown promise in the treatment of type 2 diabetes. This may be due to its effects on weight loss, in addition to decreasing insulin resistance and a favorable shift in the levels of leptin and adiponectin [32]. Patients may approach their physicians with questions regarding the implementation of intermittent fasting. In addition, physicians should be aware of the benefits of this dietary practice as a treatment for type 2 diabetes so that they may be able to help patients use this to combat the progression of their disease.

## Supplementary Information

**Additional file 1.**

## Data Availability

Data sharing is not applicable to this article as no datasets were generated or analysed during the current study.
