# Supplementary material for: Intermittent fasting: is there a role in the treatment of diabetes? A review of the literature and guide for primary care physicians
Source: Clin Diabetes Endocrinol. 2021 Feb 3;7:3. doi: 10.1186/s40842-020-00116-1 (PMC7856758; doi:10.1186/s40842-020-00116-1)
Supplement: Supplementary file 1 — Additional file 1. [file 40842_2020_116_MOESM1_ESM.docx]

Supplementary File

Inclusion/exclusion criteria for studies in Tables 1 and 2

***Trepanowski et al [28]***

*Inclusion:* Age 18-65 years, BMI 25.0 to 39.9 kg/m^2^, previously sedentary (<60 minutes/week of light activity for the 3 months prior to the study)

*Exclusion:* History of cardiovascular disease, history of type 1 or 2 diabetes, use of medications that could affect study outcomes, unstable weight for 3 months prior to beginning of the study, perimenopause or otherwise irregular menstrual cycle, pregnancy, current smoker

***Catenacci et al [19]***

*Inclusion:* Age 18-55 years, BMI >30 kg/m^2^, non-smoker, <4.5 kg weight change over the past 6 months

*Exclusion:* History of diabetes, cardiovascular disease, uncontrolled hypertension, severe dyslipidemia, on lipid lowering therapy, cancer, thyroid disease, seizures, migraines, significant renal, hepatic or gastrointestinal disorders, binge eating disorder, current depression, history of bariatric surgery, or taking medications known to affect appetite or energy metabolism. Women who were pregnant, planning pregnancy, or lactating also were excluded.

***Bhutani et al [29]***

*Inclusion:* Age 25‐65 years, BMI between 30 and 39.9 kg/m^2^, weight stable for 3 months prior to the beginning of the study (i.e., less than 5 kg weight loss or weight gain), nondiabetic, no history of cardiovascular disease, lightly active (i.e., <3 h/week of light intensity exercise at 2.5‐4.0 metabolic equivalents [METs] for 3 months prior to the study), nonsmoker, no history of bariatric surgery, and not taking weight loss, lipid, or glucose lowering medications.

*Exclusion:* Perimenopausal women and post-menopausal women were required to maintain their current hormone replacement therapy regimen for the duration of the study

***Bhutani et al [30]***

*Inclusion:* age 35–65 years, BMI between 30 and 39.9 kg/m^2^, weight stable for 3 months prior to the beginning of the study (i.e., <5 kg weight loss or weight gain), nondiabetic, no history of cardiovascular disease, normotensive (<120/<80 mm Hg) or prehypertensive (120–139/80–89 mm Hg), lightly active (i.e., <3 h/week of light intensity exercise at 2.5–4.0 metabolic equivalents for 3 months prior to the study), nonsmoker, and not taking medications that would affect study outcomes (i.e., weight loss, lipid, or glucose lowering drugs)

*Exclusion:* Perimenopausal women, women who were pregnant, and those trying to become pregnant.

***Varady et al [31]***

*Inclusion:* BMI between 20 and 29.9 kg/m^2^, age between 35 and 65 years, pre-menopausal or post-menopausal (absence of menses for more than 2 years), lightly active (< 3 h/week of light intensity exercise at 2.5 to 4.0 metabolic equivalents (METs) for 3 months prior to the study), weight stable for 3 months prior to the beginning of the study (< 4 kg weight loss or weight gain), non-diabetic, no history of cardiovascular disease, non-smoker, and not taking weight loss, lipid- or glucose-lowering medications

***Gabel et al [25]***

*Inclusion:* 18–65 years old, BMI 25.0–39.9 kg/m^2^, and previously inactive (<60 minutes/week of light activity for the 3 months prior to the study)

*Exclusion:* history of type 1 or type 2 diabetes, cardiovascular disease, were taking weight loss medications, were not weight stable for 3 months prior to the beginning of the study (> 4 kg weight loss or gain), were peri-menopausal, pregnant, or smokers

***Carter et al [32]***

*Inclusion: Age>18 years, history of type 2 diabetes, BMI >27 kg/m^2^, blood pressure <160/100, no previous history of bariatric surgery*

*Exclusion: Those who did not meet inclusion criteria, those who were pregnant or breastfeeding*

***Carter et al [24]***

*Inclusion:* Age>18 years, history of type 2 diabetes, BMI >27 kg/m^2^, blood pressure <160/100, no previous history of bariatric surgery

*Exclusion:* Those who did not meet inclusion criteria, those who were pregnant or breastfeeding

***Sundfor et al [33]***

*Inclusion: Age 21 to 70, BMI 30-45 kg/m^2^,* waist circumference ≥94/80 cm (men/women) and ≥1 additional metabolic syndrome component: circulating levels of triglycerides ≥1.7 mmol/l, HDL cholesterol ≤1.0/1.3 (men/women), blood pressure ≥130/85 mmHg or use of antihypertensive drugs or fasting glucose ≥5.6 mmol/l, and weight stability within ±3 kg during the last three months.

*Exclusion:* Diabetes if treated with insulin or incretin analogues, bariatric surgery, use of anti-obesity drugs or other drugs affecting body weight, eating disorder, or psychiatric illness, or alcohol or drug abuse that could contribute to difficulties with study procedures

***Corley et al [34]***

*Inclusion:* Age >18 years with Type 2 diabetes who were taking medication for diabetes, including metformin and/or any combination of hypoglycemic agents, HbA1c concentration in the range 50–86 mmol/mol (6.7 to 10.0%), BMI 30–45 kg/m2

*Exclusion:* History of Type 1 diabetes, weight change of >5 kg in the preceding 3 months, diagnosis of an eating disorder, pregnancy or planning pregnancy, blood pressure > 180/100 mmHg despite medical therapy, previous bariatric surgery, and any significant medical condition which, in the view of study investigators, would make recruitment to the study inappropriate

***Moro et al [35]***

*Inclusion:* Subjects must have performed resistance training continuously for at least 5 years (training 3–5 days/week with at least 3 years experience in split training routines), currently engaged in regular resistance training, must be life-long steroid free, and have no clinical problems that could be aggravated by the study procedures.

***Sutton et al [36]***

*Inclusion:* Age 35 -70 years, male gender, A1C 5.5-6.4%, 2 hour glucose tolerance test 140-199 mg/dL, BMI 25-50 kg/m^2^

***Hutchison et al [37]***

*Inclusion:* age 30 to 70 years, waist circumference ≥ 102 cm, weight stability (within 5% of screening weight) for > 6 months prior to study entry, nonsmoker, being sedentary or lightly active (i.e., took part in < 2 moderate‐ to high‐intensity exercise sessions per week), consuming < 140 g/wk of alcohol, no prior diagnosis of type 2 diabetes, not taking antihyperglycemic medication, and no personal history of cardiovascular disease, eating disorders, or major psychiatric disorders

***Cienfuegos et al [38]***

*Inclusion:* BMI 30.0 to 49.9 kg/m^2^, age 18 to 65 years; sedentary (light exercise less than 1 h per week) or moderately active (moderate exercise 1 to 2 h per week); weight stable for 3 months prior to the beginning of the study (gain or loss <4 kg)

*Exclusion:* History of diabetes mellitus, use of medications that could affect study outcomes, night shift workers, perimenopausal or otherwise irregular menstrual cycle, pregnant or trying to become pregnant, and current smokers

***Furmli et al [23]***

*Inclusion:* History of Type 2 Diabetes, requiring at least 70 units of insulin daily

***Hoddy et al [20]***

*Inclusion:* BMI 30 to 39.9 kg/m^2^, age 25 to 65 years, pre‐menopausal or post‐menopausal (absence of menses for more than 2 years), lightly active (<3 h/week of light‐intensity exercise at 2.5‐4.0 metabolic equivalents [METs] for 3 months prior to the study), weight stable for 3 months prior to the beginning of the study (<4 kg weight loss or weight gain), non‐diabetic, no history of cardiovascular disease (myocardial infarction or stroke), non‐smoker, and not taking weight loss, lipid‐ or glucose‐lowering medications

***Lichtash et al [39]***

*Inclusion:* Normal weight, history of type 2 diabetes, failed medical therapy
